# Effect of Cadmium on Growth, Bacoside A, and Bacopaside I of *Bacopa monnieri* (L.), a Memory Enhancing Herb

**DOI:** 10.1155/2014/824586

**Published:** 2014-01-30

**Authors:** Poonam Gupta, Sayyada Khatoon, P. K. Tandon, Vartika Rai

**Affiliations:** ^1^Department of Botany, University of Lucknow, Lucknow, Uttar Pradesh 226007, India; ^2^Pharmacognosy and Ethnopharmacology Division, CSIR-National Botanical Research Institute, Lucknow, Uttar Pradesh 226001, India

## Abstract

*Bacopa monnieri* (L.) is an important medicinal plant mainly known as a memory enhancing herb. It is important to see the effect of metal pollution on its active constituents. In this context, efforts have been made to observe the effect of Cd on the triterpenoid saponins bacoside A and bacopaside I in this plant. The influence of the metal on growth parameters like protein, chlorophyll content, and biomass has also been observed. It is interesting to note that the bacoside A and bacopaside I gradually increased by the Cd treatment up to 10 *μ*M and then decreased at higher concentrations, that is, 50 and 100 *μ*M, but the concentration of these components was more in all the treated plants as compared to control. On the contrary, protein, chlorophyll content, and biomass decreased with the increase in metal concentration and exposure duration due to metal toxicity.

## 1. Introduction

*Bacopa monnieri* (L) Pennell (Fam. Scrophulariaceae) is growing in the marshy places, which may be natural, or manmade with high possibility of heavy metal contamination as manmade marshy places are generally created in industrial areas where industrial effluents were discharged with high metal content. In such places one can see the luxuriant growth of *B. monnieri*. In this context, it becomes necessary to check the effect of heavy metals on general growth and active constituents of the plant, because demand of this plant is quite high in market and plant collectors collect it without considering polluted or nonpolluted site. Cadmium is one of the heavy metals that is of great concern in the environment because of its toxicity to all the plants, animals, and human beings. Cd enters in the environment through industrial waste from processes such as electroplating, manufacturing of plastics, mining, paint pigments, alloy preparation, and batteries that contain cadmium [1, 2]. Cadmium is also used for luminescent dials, in photography and rubber curing, and as fungicides [1]. The most likely origin of the excess Cd is from heavy applications of cheap, contaminated phosphate fertilizers [3, 4]. It is considered to be highly mutagenic and designated as human carcinogen by International Agency for Research on Cancer [5, 6]. It is retained for many years in the human body and may induce chronic toxicity [7, 8]. Elevated levels of Cd in humans can cause kidney damage, and low levels of Cd in the diet are linked renal to dysfunction. Other diseases associated with Cd exposure are pulmonary emphysema and the notorious Itai-Itai (“ouch-ouch”) disease [9]. Therefore, effective and economical techniques are needed to remediate Cd-contaminated soils.

*B. monnieri* is a small creeping herb, commonly growing in marshy places up to an altitude of 1500 m. It is an important Ayurvedic drug and traditionally it is reported to be used in skin diseases, fever, inflammation, anaemia, urinary disorder, and psychiatric disorders [10]. It is also considered to be cardiotonic, a potent nervine tonic, and useful for asthma, hoarseness, insanity, and epilepsy [11]. Ethnobotanically the leaves are used in speech disorders [12]; in premature ejaculation [13]; flatulence [14]; abdominal pain [15]; cough and cold [16–18], and leaf juice is used in rheumatism [19, 20] revitalizer of intellectual faculty [21]. The major therapeutically important chemical constituents of this plant are triterpenoid saponins bacosides. The pharmacological [22, 23] and clinical studies [24, 25] on the extract and bacoside A and B have been published. The extracts have economic importance too as it is widely available in the international nutraceutical markets.

Although some reports are available on the accumulation and toxicity of heavy metals in *B. monnieri* [26–32], but no report is available on the effect of Cd on the active principles of *B. monnieri*. Keeping this in mind an attempt was made to check the effect of Cd on growth and active constituents of *B. monnieri,* that is, bacoside A and bacopaside I.

## 2. Material and Methods

Plants of *Bacopa monnieri* were collected from unpolluted sites and grown in hydroponic cultures for several months in the field laboratory. The healthy vegetative clones of *B. monnieri* were further acclimatized in 3% Hoaglands' nutrient medium [33] under standard physiological conditions providing 16 hrs light period (114 *μ*mol M^−2^ s^−1^) and 8 hrs dark photoperiod, 26 ± 2°C for 6 weeks.

Different concentrations of Cd, that is, 5 *μ*M, 10 *μ*M, 50 *μ*M, and 100 *μ*M, were prepared in 3% Hoagland's solution using AR grade cadmium chloride (CdCl_2_). Only 3% Hoagland's solution without addition of Cd served as control. Young plants of *B. monnieri* with 14–16 internodes and 2-3 basal rooted nodes were incorporated in each concentration under the abovementioned standard physiological conditions. The whole experiment was repeated at least three times. Aeration was provided to all the plants. The plants were harvested after 48, 96, and 168 hrs for estimation of metal uptake, protein and chlorophyll content, and biomass (fresh weight). Bacoside A and bacopaside I contents were measured in control, 5 *μ*M 10 *μ*M, 50 *μ*M, and 100 *μ*M Cd concentrations after one week.

Biomass was calculated on fresh weight basis using electronic balance. The fresh plant was used for the estimation of chlorophyll content. 100 mg fresh weight of the plant was extracted in 80% chilled acetone and estimated following the method of Arnon [34]. Protein content in the plants of *B. monnieri* was estimated by the method of Lowry et al. [35] using bovine serum albumin (BSA) as a standard. For metal uptake the harvested plant material was washed with deionized water, weighed and dried at 70°C for 48 hrs, and digested with HNO_3_ : HCLO_4_ (10 : 1 v/v mixture). Cd was estimated using Perkin Elmer 2380 Atomic Absorption Spectrophotometer (detection limit of Cd: 0.0005 ppm).

For quantitative thin layer chromatography (TLC) for bacoside A and bacopaside I, ten grams of powdered plant material of control and Cd treated plant was extracted with 50 mL methanol on water bath consecutively three times. Extracts were filtered and concentrated at low temperature and reduced pressure. Bacoside A and bacopaside I standard solutions of 1 mg/mL concentration were prepared by dissolving the standards in methanol. 10 *μ*L of standards and plant extracts was applied on HPTLC precoated silica gel plates (E-MERCK F_254_) with the help of CAMAG Linomat V Applicator. The plates were developed to a distance of 9.0 cm in the solvent system Chloroform : Methanol : Water (7 : 3 : 0.5) in previously saturated twin trough chamber (CAMAG). The plates were scanned at the wave length 500 nm using CAMAG TLC Scanner 3 with software winCATS. Photographs of TLC plates were taken by the CAMAG Reprostar-3.

### 2.1. Statistical Analysis

The experiment was set up as randomized block design. To confirm the variability of data and validity of results all the data were subjected to analysis of variance (ANOVA) followed by Newman Keuls' test for individual comparison. Comparison between control and treatment was done by LSD test [36].

## 3. Results and Discussion

Higher Cd treatments, that is, 50 and 100 *μ*M, produce visible morphological symptoms like decay of basal portion of shoot and leaves due to Cd toxicity after one week. The intensity of Cd toxicity enhanced with increase in the exposure duration. Browning and stunting of roots were also observed in 50 *μ*M Cd and above after one week. The significant decrease in the biomass of plant is also observed at higher concentrations (*P* < 0.05) (Table 1). Decrease in biomass also confirms that Cd affects the growth of plants at higher concentrations. This is in accordance with the studies of Haag-Kerwer et al., [37] in which Cd accumulation results in the decrease in growth rate and reduction in transpiration of mustard plants. A significant (*P* < 0.01) decrease in the chlorophyll content with the increase in the metal concentration (Table 2) was observed in the present study. This might be due to interaction of Cd with –SH group of various enzymes involved in the chlorophyll biosynthesis [38]. The decrease in the protein content of the plant with increase in the metal concentration was also highly significant (*P* < 0.01) (Table 3). A significant decrease in the protein content may be due to Cd-induced oxidation of proteins mediated by H_2_O_2_ and due to increased proteolytic activity which has been proposed as an index of oxidative stress [39].

The uptake of Cd by the plant was doze and duration dependent and also highly significant (*P* < 0.01). At the lowest ambient concentration of 5 *μ*M the uptake was as high as 169.91 *μ*g/g dry wt, within 48 hrs. However maximum uptake was 1779.70 *μ*g/g, after 168 h observed in 100 *μ*M concentrations (Table 4). Although Cd is a highly toxic metal for the growth and development of plant but it is highly accumulated by aquatic macrophytes, which has also been previously reported by various workers [40–44]. Accumulation of cadmium by *B. monnieri* has also been reported earlier by Sinha and Chandra [26]; Sinha [29]; Ali et al. [30]; and Singh et al. [32]. It can be said with these results that *B. monnieri* can work as potential accumulator of Cd and can be used in the phytoremediation. Phytoremediation is the use of green plants to detoxify a degraded or polluted environment [45]. The main advantages of phytoremediation are that the procedure is carried out in situ and it is inexpensive compared to other technologies for remediation [45]. Another important advantage of phytoremediation is that soils retain their fertility after metal removal.

**Figure 1:**
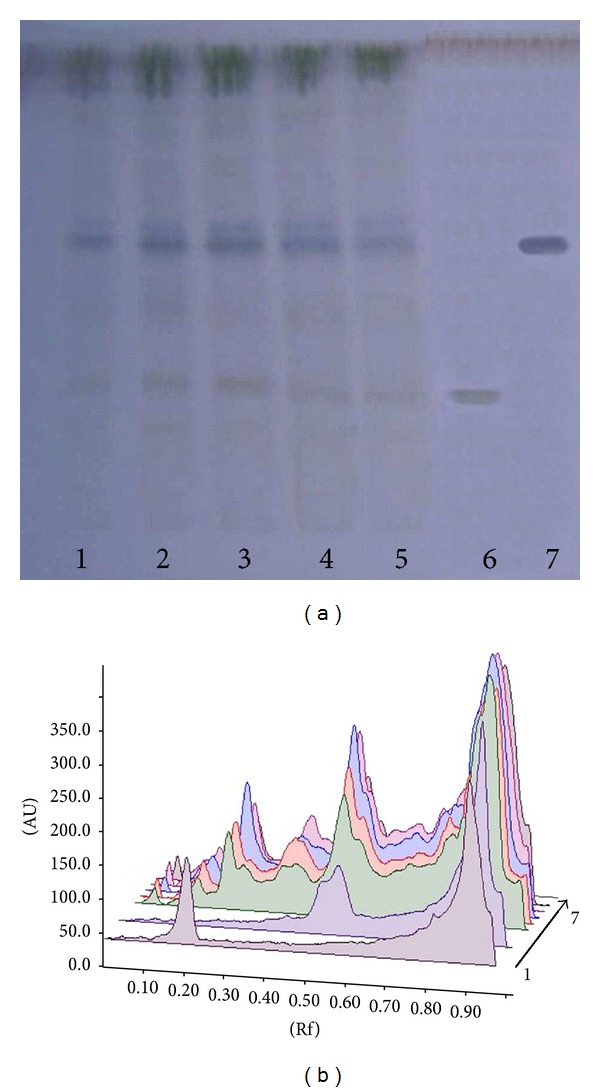
TLC of methanolic extract of control and Cd treated plants of *Bacopa monnieri*. (a) Fingerprint profile, 1: control, 2: 5 *μ*M Cd, 3: 10 *μ*M Cd, 4: 50 *μ*M Cd, 5: 100 *μ*M, 6: bacoside A, and 7: bacopaside I. (b) Densitometric scan at 500 nm.

The TLC fingerprint profile showed the increase in the content of the bioactive compounds, that is, bacoside A and bacopaside I in all Cd treated plants (Figure 1). The retention factor values and the colour of components present in *B. monnieri* extracts are shown in Table 5.

**Figure 2:**
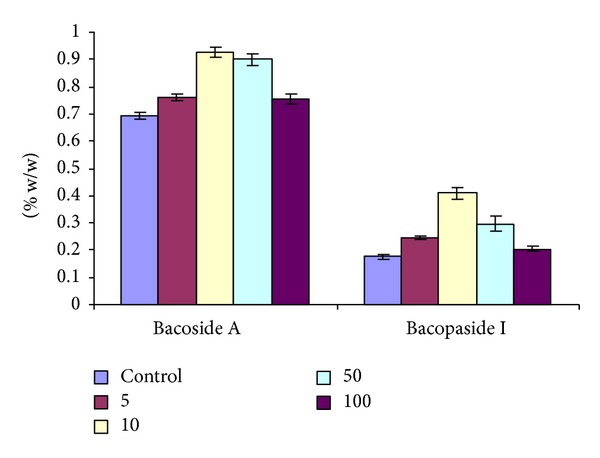
Effect of cadmium on the bacoside A and bacopaside I content in *B. monnieri*.

It is a well-known fact that secondary metabolites are formed under various stresses as a defense mechanism [46]. In the present study it is interesting to note that the aforesaid compounds were gradually increased by the Cd treatment up to 10 *μ*M and then decreased at higher concentrations, that is, 50 *μ*M and 100 *μ*M (Figure 2). This indicates that the synthesis of secondary metabolites enhances initially up to a certain limit due to abiotic stress and then decreases due to Cd toxicity in higher concentrations. There are several examples available where plants synthesize and accumulate secondary metabolites upon treatment with heavy metals [47–50].

Thus, it is clear from this study that *B. monnieri* may become a very important plant for phytoremediation and remove Cd from the polluted site, and it is a good indication that its active constituents increase in this condition. Pharmaceutical companies may use these plants for extracting its active compounds, even if grown on polluted sites.

## Figures and Tables

**Table 1:** Effect of cadmium on biomass (g fw) in *B. monnieri *at different concentrations and exposure periods.

Cd concentrations (*μ*M)	Exposure periods (h)			
	0	48	96	168
0 *μ*M	1.81 ± 0.14	1.87 ± 0.17	2.07 ± 0.25	2.14 ± 0.24
5 *μ*M	2.03 ± 0.11	2.06 ± 0.12	2.08 ± 0.03	2.17 ± 0.03
10 *μ*M	1.75 ± 0.52	1.75 ± 0.52	1.79 ± 0.52	1.86 ± 0.57
50 *μ*M	1.53 ± 0.42	1.52 ± 0.42	1.20 ± 0.23^A^	1.13 ± 0.16^a^
100 *μ*M	1.31 ± 0.19	1.31 ± 0.19	0.98 ± 0.07^B^	0.94 ± 0.06^b^

All values are means of triplicate ± SD.

^A^denote the significance (*P* < 0.05) at 50 *μ*M cadmium as compared to control after 96 hours.

^B^denotes the significance (*P* < 0.01) at 100 *μ*M cadmium as compared to control after 96 hours.

^a^denotes the significance (*P* < 0.05) at 50 *μ*M cadmium as compared to control after 168 hours.

^b^denotes the significance (*P* < 0.05) at 100 *μ*M cadmium as compared to control after 168 hours.

**Table 2:** Effect of cadmium on the chlorophyll content (mg g^−1^ fw) in *B. monnieri *at different concentrations and exposure periods.

Cd concentration (*μ*M)	Exposure periods (h)		
	48	96	168
0	1.23 ± 0.02	1.36 ± 0.04	1.37 ± 0.03
5 *μ*M	1.25 ± 0.03^A^	1.32 ± 0.02^B^	1.36 ± 0.01^C^
10 *μ*M	1.24 ± 0.04^A^	1.22 ± 0.02^B^	1.10 ± 0.02^C^
50 *μ*M	1.21 ± 0.01^A^	0.99 ± 0.01^B^	0.74 ± 0.02^C^
100 *μ*M	1.00 ± 0.02^A^	.72 ± 0.01^B^	0.51 ± 0.04^C^

All values are means of three replicates ± SD; LSD *P* < 0.01.

^A^denotes significance (*P* < 0.01) at different Cd concentrations as compared to control after 48 hours.

^B^denotes significance (*P* < 0.01) at different Cd concentrations as compared to control after 96 hours.

^C^denotes significance (*P* < 0.01) at different Cd concentrations after 168 hours.

**Table 3:** Effect of Cd on the protein content (mg g^−1^ fw) of *B. monnieri * at different concentrations and exposure periods.

Cd concentration (*μ*M)	Exposure periods (h)		
	48	96	168
Control 0	12.33 ± 0.04	12.33 ± 0.01	12.50 ± 0.08
5 *μ*M	11.99 ± 0.01^A^	11.09 ± 0.01^A^	10.21 ± 0.01^A^
10 *μ*M	11.80 ± 0.09^A^	11.54 ± 0.20^A^	9.25 ± 0.01^A^
50 *μ*M	^A^10.07 ± 0.03^A^	8.09 ± 0.01^A^	7.06 ± 0.03^A^
100 *μ*M	9.66 ± 0.07^A^	7.51 ± 0.01^A^	5.10 ± 0.01^A^

All the values are means of three replicates, LSD *P* < 0.01; ^A^denotes significance (*P* < 0.01) at different Cd concentrations and exposure periods as compared to control.

**Table 4:** Accumulation of Cd (*µ*g g^−1^ dw) in* B. monnieri* at different concentrations and exposure periods.

Cd concentrations (*μ*M)	Exposure periods (h)		
	48	96	168
0.0	ND	ND	ND
5 *μ*M	169.91 ± 3.05	280.35 ± 25.34	330.07 ± 31.58
10 *μ*M	290.32 ± 19.08	667.62 ± 47.13	718.56 ± 2.74
50 *μ*M	584.47 ± 51.55	1140.16 ± 33.92	1729.21 ± 50.23
100 *μ*M	971.84 ± 20.16	1303.07 ± 84.41	1779.91 ± 49.07

All values are means of triplicate ± SD; LSD *P* < 0.01.

**Table 5:** *R*_*f*_ values and the colour of components present in *B. monnieri* extracts.

Colour	*R*_*f*_
Yellowish brown	0.20
Brown (I)	**0.26 bacopaside** I
Yellow	0.39
**Purple blue (a)**	**0.57 bacoside A**
**Blue (a)**	**0.60 A**
Light blue	0.67
Light blue	0.71
Light blue	0.78
Greenish	0.82

Bold fonts refer to *R*_*f*_s of Bacoside and Bacopaside.
